# Horizontal Heart Orientation as a Mechanistic Contributor to Platypnea–Orthodeoxia Syndrome in Patent Foramen Ovale

**DOI:** 10.1002/ccr3.72642

**Published:** 2026-04-28

**Authors:** Bijeta Keisham, Sanchit Duhan, Uday B. Kanakadandi, Osama Alsara, Naveed A. Adoni, Sanjay S. Mehta, Andrea Brasch, Haseeb Basha, Vishesh Paul

**Affiliations:** ^1^ Department of Internal Medicine Shandong Second Medical University Weifang Shandong China; ^2^ Department of Cardiology, Carle Foundation Hospital University of Illinois Urbana‐Champaign Urbana Illinois USA; ^3^ Department of Cardiology University of Illinois Urbana‐Champaign Urbana Illinois USA; ^4^ Department of Pulmonary Medicine and Critical Care, Carle Foundation Hospital University of Illinois Urbana‐Champaign Urbana Illinois USA

**Keywords:** orthodeoxia, patent foramen ovale, PFO, platypnea, POS, septal occluder

## Abstract

Altered cardiac orientation in the presence of a patent foramen ovale (PFO) can contribute to platypnea–orthodeoxia syndrome (POS) and unexplained hypoxemia. In such cases, consideration of PFO closure may lead to significant clinical improvement.

## 1 Introduction

Platypnea–orthodeoxia syndrome (POS) refers to dyspnea and hypoxemia that worsen in the upright position compared with the recumbent position. A significant drop in oxygen saturation is defined as a PaO_2_ decrease of > 4 mmHg or a SaO_2_ decrease of > 5% when moving from supine to upright position [1]. These changes are usually reversible by returning to the supine position. The causes of POS include intracardiac shunts, pulmonary arteriovenous shunts, and parenchymal ventilation–perfusion mismatch (as in chronic obstructive pulmonary disease or interstitial lung disease) [2]. The first reported case of POS, in 1949, occurred in a patient with a post‐traumatic intrathoracic arteriovenous shunt [3]. Since then, several etiologies and exacerbating factors have been identified, but the pathophysiology remains incompletely understood and primarily supported by observational data. We present a rare case of POS to further our understanding of the disease process and to aid in clinical diagnosis.

## 2 Case History/Examination

A man in his 70s presented to the outpatient pulmonology clinic with worsening shortness of breath. His past medical history included asthma, obstructive sleep apnea (compliant with nocturnal CPAP), hypersensitivity pneumonitis, and chronic respiratory failure on 3 L of home oxygen.

For the past month, he had experienced progressive dyspnea despite increasing his home oxygen to 6 L, with desaturations to 70% on his home pulse oximeter. One month earlier, he could walk 100 ft without difficulty, but he was now limited to a few feet. He denied recent infections or other associated symptoms.

He was a former smoker (10.5 pack‐years, quit 50 years ago), an army veteran with exposure to Agent Orange, and a farmer. He had no family history of significant cardiac or pulmonary disease. He was adherent to his medications, including budesonide–formoterol, tiotropium, diltiazem, and lisinopril.

He was sent to the emergency department (ED) for further evaluation.

## 3 Differential Diagnosis, Investigations and Treatment

**FIGURE 1:**
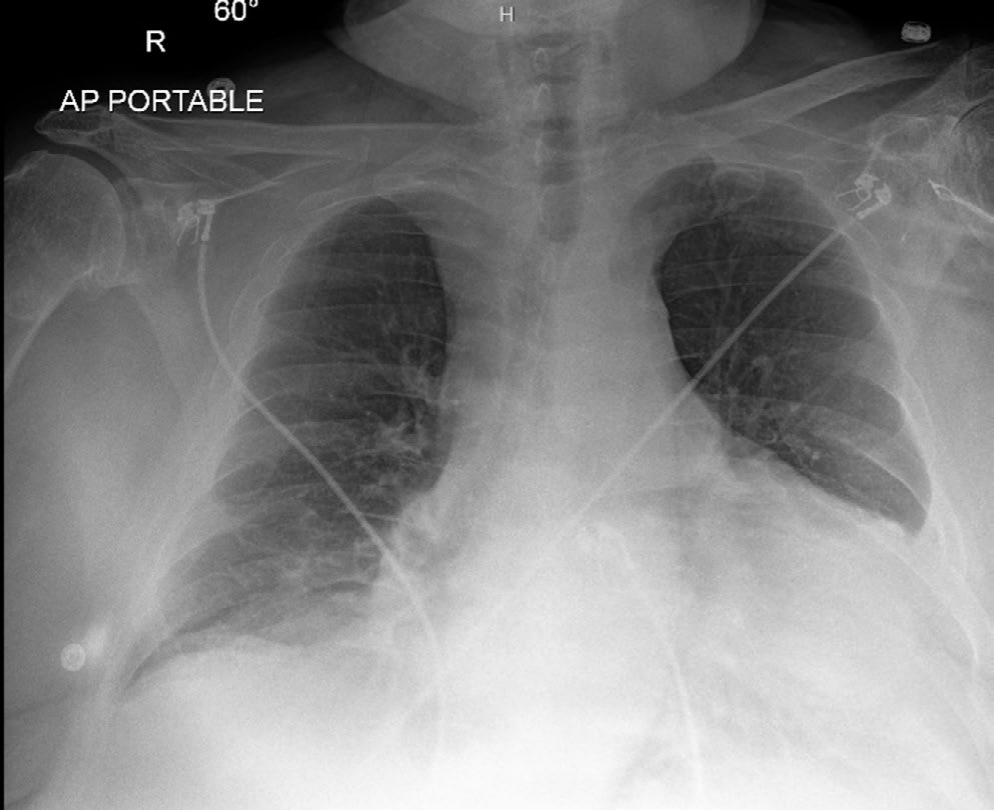
Chest x‐ray AP view. R = right.

**FIGURE 2:**
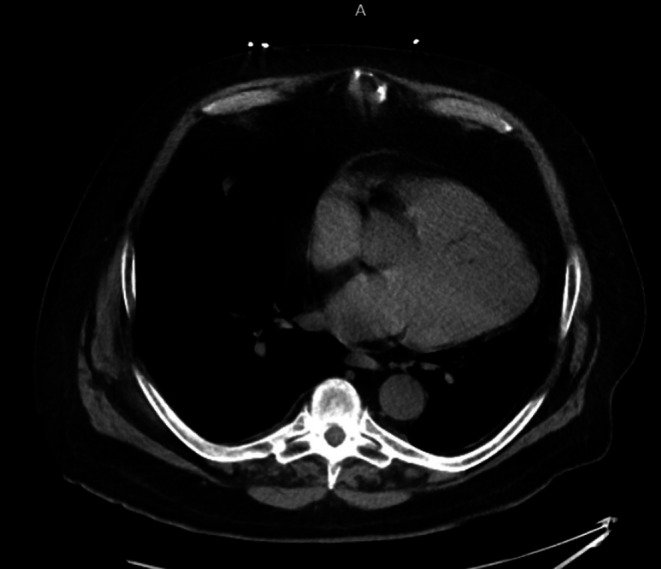
CT chest without contrast.

**FIGURE 3:**
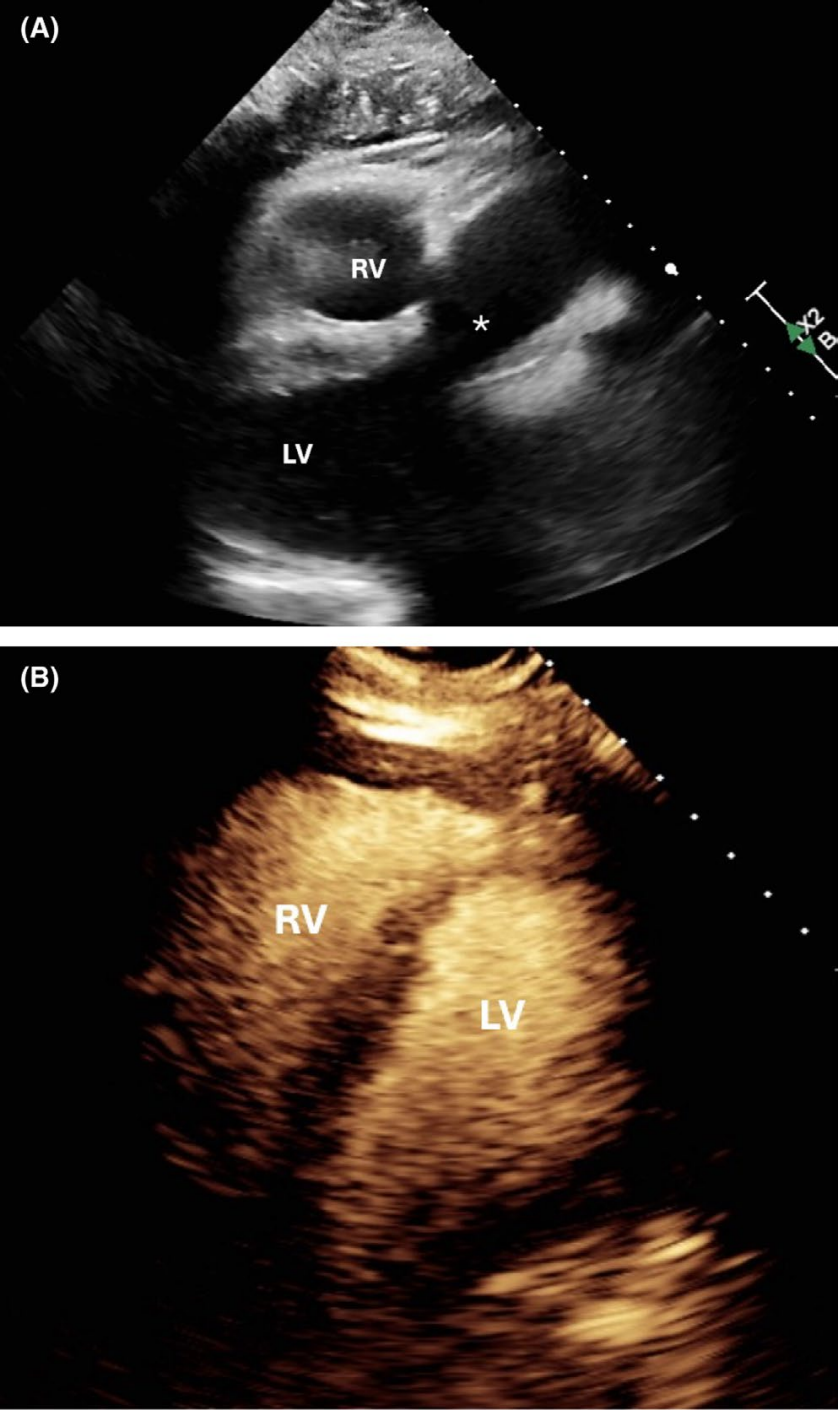
(A) Transthoracic echocardiogram (TTE) (parasternal long axis view) showing overriding aorta (marked by *). LV = left ventricle, RV = right ventricle. (B) Contrast images. LV, left ventricle; RV, right ventricle.

In the ED, he was hypoxemic (SpO_2_ 85%–87% on 10 L nasal cannula), tachypneic (20 breaths/min), with wheezing, decreased breath sounds, and truncal cyanosis. His other vital signs included normal temperature (36.3°C), blood pressure 111/75 mmHg, and pulse rate (72 beats/min). Laboratory workup—including CBC, CMP, BNP, troponin, lactate, and D‐dimer—was unremarkable. Chest radiography (Figure 1) and CT (Figure 2) showed cardiomegaly, bilateral pleural effusions, and an unusually horizontal cardiac silhouette. Echocardiography (Figure 3) revealed a severely enlarged right ventricle, moderate–severe right ventricular systolic dysfunction, and preserved left ventricular function. An overriding aorta was incidentally noted.

**FIGURE 4:**
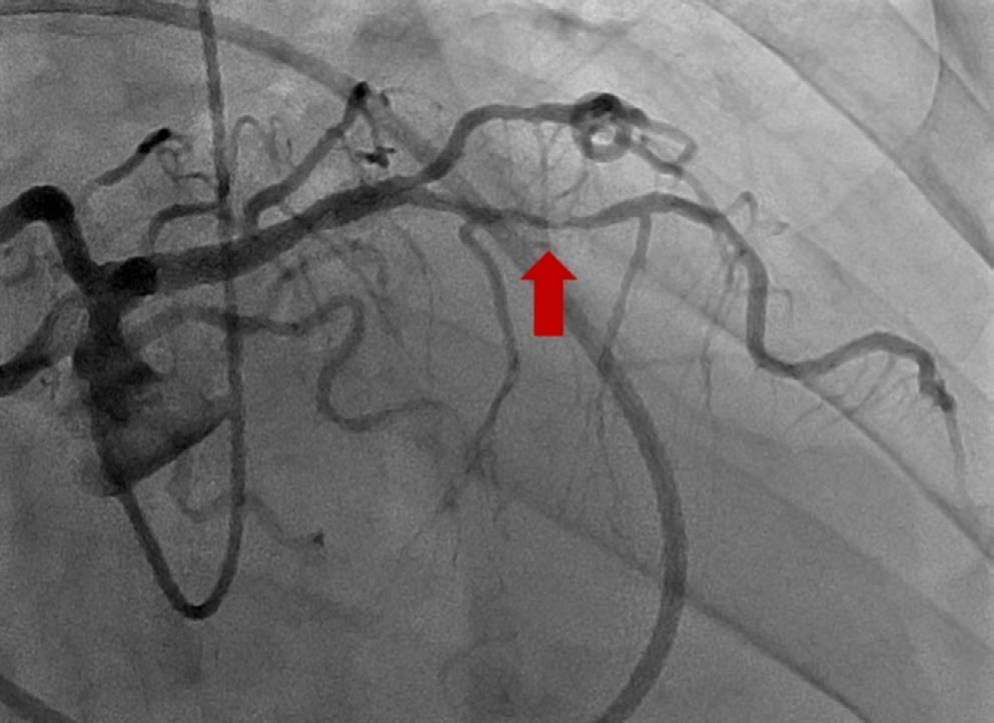
Left heart catheterization (LHC) showing mid‐left anterior descending stenosis (arrow).

Despite treatment for suspected asthma and heart failure exacerbation, his respiratory status did not improve. After discussion with the pediatric cardiology team, he underwent right and left heart catheterization (Figure 4 and Table 1) to assess for intracardiac shunting and ischemia. A 90% mid–left anterior descending artery stenosis was identified and successfully stented. Normal right and left heart filling pressures and a preserved cardiac index were observed. No intracardiac shunt was documented (Qp: Qs 1.04).

**TABLE 1:** Right heart catheterization hemodynamics.

Rhythm	Sinus rhythm
Average heart rate during the procedure	75 bpm
Right atrial pressure	6 mmHg
Right ventricular pressure	19/0/3 mmHg
Pulmonary artery pressure	20/9 mmHg with a mean of 15 mmHg
Pulmonary capillary wedge pressure	10 mmHg
Transpulmonary gradient	5 mmHg
Pulmonary vascular resistance	1.03 Wood Units
Systemic vascular resistance	1392 dsc‐5
*Oxygen saturations*	
SVC saturation	58.8%
RA saturation	61.5%
Pulmonary artery	61.6%
Aorta	86.1%
Qp/Qs	1.04
Indirect Fick Cardiac Output	4.82 L/min
Indirect Fick Cardiac Index	2.1 L/min/m^2^

**FIGURE 5:**
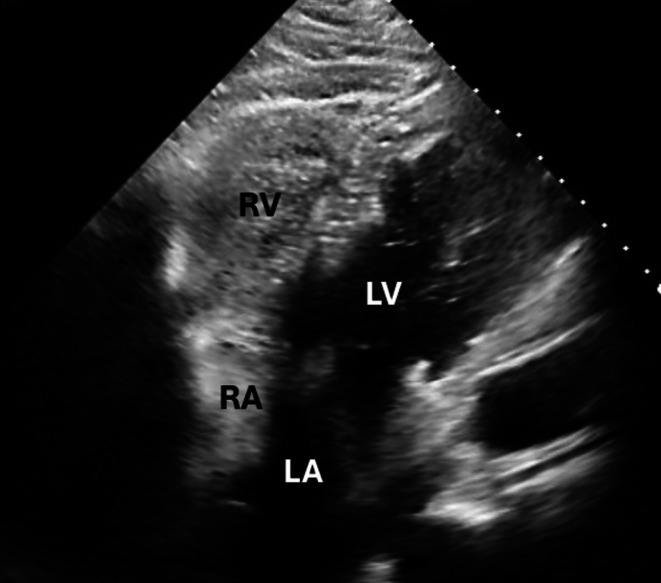
Transthoracic echocardiogram (TTE) with agitated saline showing right‐to‐left shunting. LA, left atrium; LV, left ventricle; RA, right atrium; RV, right ventricle.

**FIGURE 6:**
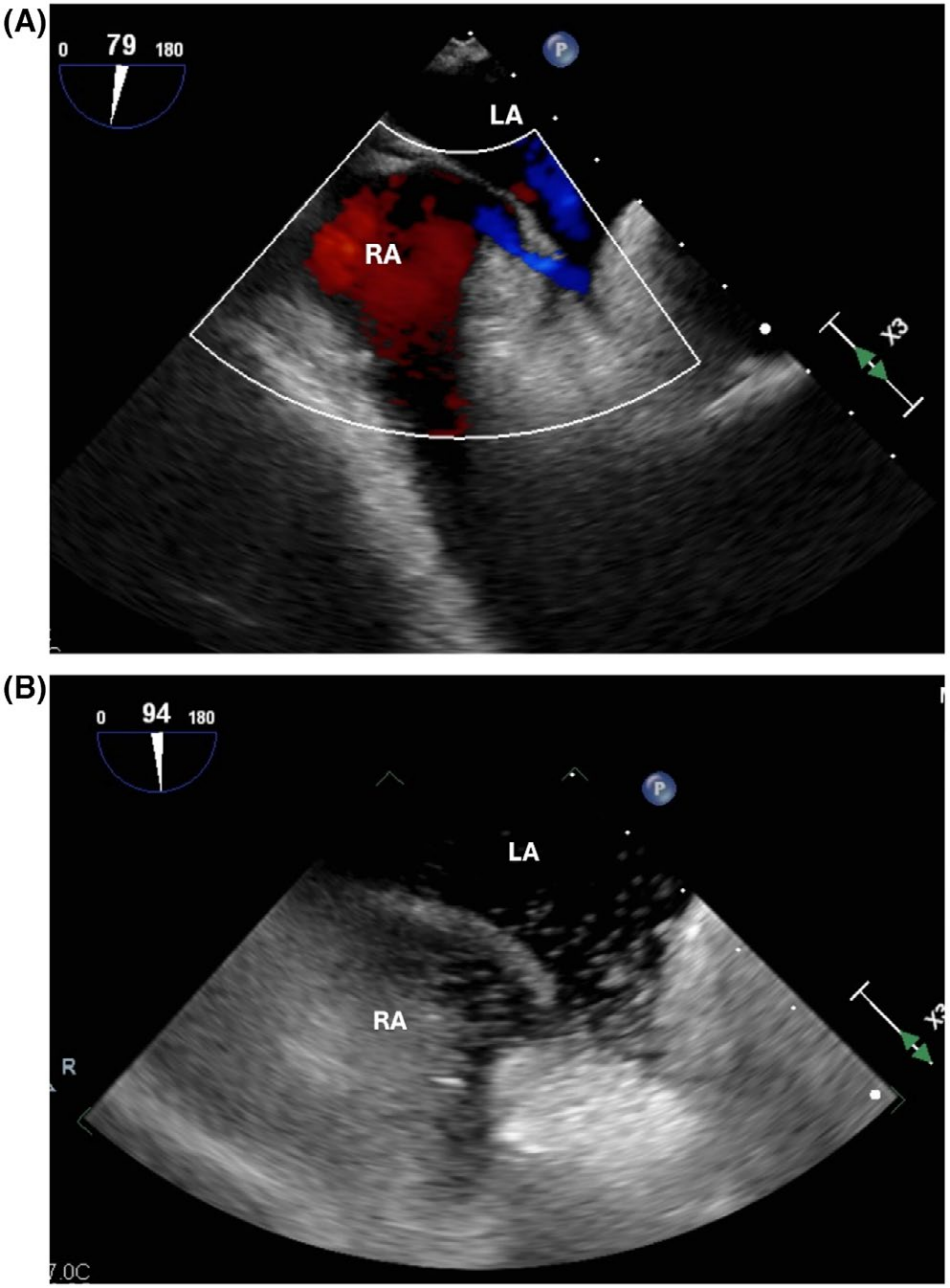
(A) Transesophageal echocardiogram (TEE) with color doppler showing patent foramen ovale (PFO). LA, left atrium; RA, right atrium. (B) TEE with agitated saline confirming right‐to‐left shunting via PFO. LA, left atrium; RA, right atrium.

His hypoxia worsened, and on hospital day 5, he required 100% FiO_2_ with SpO_2_ ranging from 80% to 82% on high‐flow nasal cannula. Platypnea was noted, with SpO_2_ values as follows: right lateral decubitus 82%–83%, left lateral decubitus 88%–89%, and sitting upright 77%–78%. Given persistent suspicion for intracardiac shunting, a transthoracic echocardiogram with agitated saline (Figure 5) was performed, suggesting right‐to‐left interatrial shunting. Transesophageal echocardiography (Figure 6) confirmed significant right‐to‐left shunting through a patent foramen ovale (PFO).

**FIGURE 7:**
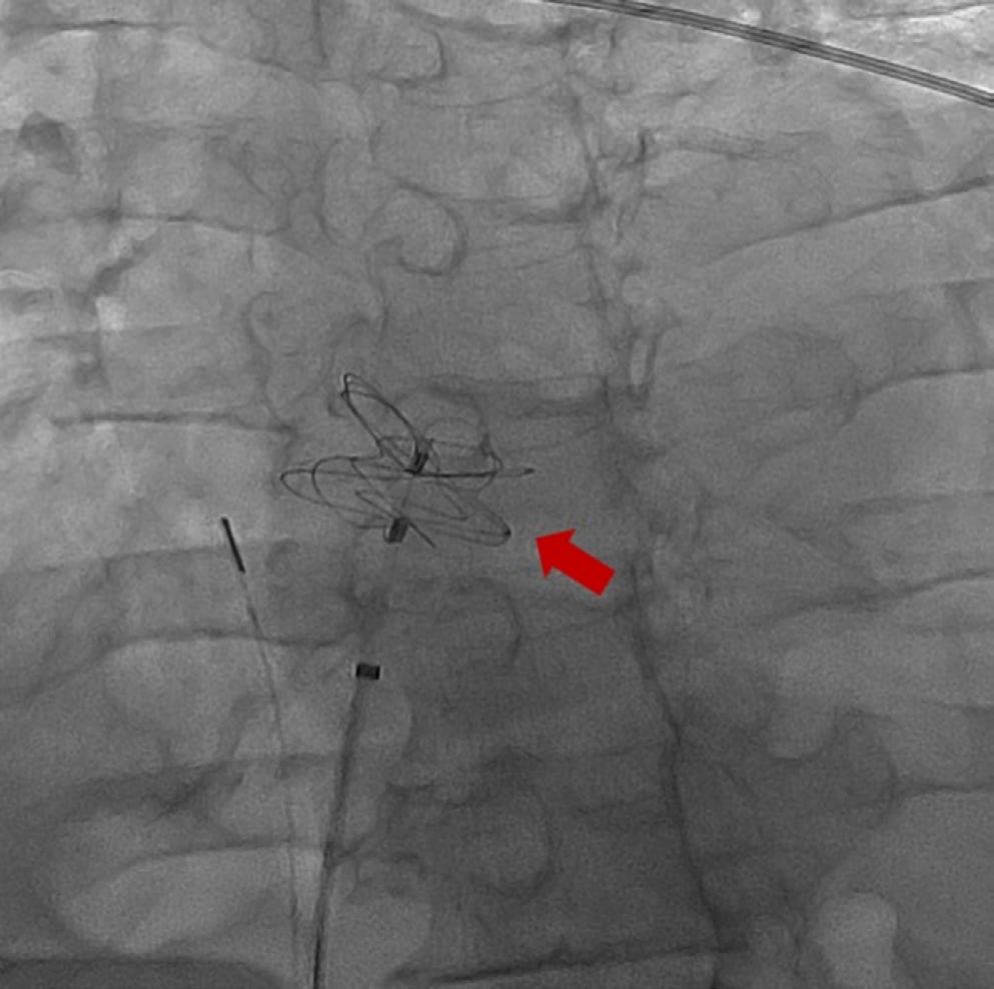
Septal occlude device (arrow).

Evidence for PFO closure in POS is limited. The 2022 SCAI guidelines provide a conditional recommendation for closure in these patients, citing very low certainty of evidence. After counseling about the uncertain benefit, the patient elected to proceed with closure. Intraoperatively, the defect measured 0.9 cm and was closed with a 37 mm GORE CARDIOFORM Septal Occluder (Figure 7) on hospital day 11.

## 4 Outcome and Follow‐Up

The following day, he was able to rest comfortably in a chair with SpO_2_ > 90% on room air. His symptoms improved markedly, cyanosis resolved, and ambulatory capacity increased. He was discharged on day 14. At follow‐up visits at 1 and 4 weeks, he remained off supplemental oxygen, had an SpO_2_ of ~94%, and reported a substantial improvement in quality of life.

## 5 Discussion

Cases of POS due to PFO have been reported in middle‐aged and elderly patients [4, 5, 6, 7]. Approximately 2% of individuals with a PFO develop POS [7]. It is unclear why the syndrome manifests later in life despite PFO being a congenital defect.

The pathophysiology of POS in the setting of PFO is linked to atrial stretch and alterations in blood flow. In the supine position, the right atrium is tense, and blood from the inferior vena cava (IVC) flows into the atrial cavity, keeping the PFO functionally small. In the upright position, the atrial septum relaxes and IVC flow is redirected toward the defect, enlarging the PFO and increasing the shunt [8, 9]. As seen in this case, the diagnosis can be missed during a right heart catheterization performed in the supine position, as desaturation occurs only when the patient is upright. This positional desaturation is an important diagnostic clue.

Most patients remain asymptomatic because left atrial pressure typically exceeds right atrial pressure by 5–8 mmHg, functionally closing the septum. However, elevated right atrial pressure or anatomical distortion can precipitate symptoms [10, 11, 12]. In some cases, deformation or displacement of the atrial septum changes the shape and compliance of the right atrium, altering PFO position and increasing shunting. Direct alignment of IVC flow with the PFO can further exacerbate right‐to‐left shunting [11, 12]. Distortion of the interatrial septum has also been associated with dilation of the proximal ascending aorta [12].

In a recently published case of POS in a patient with small bowel obstruction (SBO), it was hypothesized that the increased intra‐abdominal pressure associated with the SBO led to increased intra‐thoracic pressure and elevated right atrial pressure, exacerbating right‐to‐left shunting [13]. We speculate that age‐related tissue degeneration and PFO enlargement, combined with horizontal displacement of the heart—possibly due to abdominal obesity—aligned the IVC jet with the PFO in our patient, precipitating POS.

The 2022 SCAI guidelines recommend PFO closure in POS without prior PFO‐associated stroke, but the recommendation is conditional due to very low‐quality evidence. Data from observational studies suggest improvements in oxygenation and quality of life, but potential risks include atrial fibrillation, mortality, and procedural complications [14, 15, 16]. Given the marked improvement in our patient and the growing safety of percutaneous closure, future guidelines may provide a stronger recommendation.

## Author Contributions

**Bijeta Keisham:** resources, software, validation, visualization, writing – original draft, writing – review and editing. **Sanchit Duhan:** conceptualization, resources, visualization, writing – original draft, writing – review and editing. **Uday B. Kanakadandi:** supervision, writing – review and editing. **Naveed A. Adoni:** resources, supervision. **Osama Alsara:** supervision. **Sanjay S. Mehta:** supervision. **Andrea Brasch:** supervision. **Haseeb Basha:** supervision. **Vishesh Paul:** supervision, validation, writing – review and editing.

## Funding

The authors have nothing to report.

## Consent

Author has obtained written informed consent from patient.

## Conflicts of Interest

The authors declare no conflicts of interest.

## Data Availability

No new data is generated.
